# Severity of misophonia symptoms is associated with worse cognitive control when exposed to misophonia trigger sounds

**DOI:** 10.1371/journal.pone.0227118

**Published:** 2020-01-16

**Authors:** Emily C. Daniels, Andrew Rodriguez, Darya L. Zabelina

**Affiliations:** Department of Psychological Science, University of Arkansas, Fayetteville, AR, United States of America; University of Regensburg, GERMANY

## Abstract

The present study aimed to investigate the extent to which the severity of misophonia symptoms is linked with cognitive control under misophonia symptom-provocation circumstances in the general population sample. Participants (N = 79) completed a measure of cognitive control–a Stroop color naming task, which consists of congruent and incongruent stimuli, and requires inhibition of a prepotent response (reading a word) in the service of a less predominant response (naming a color), while listening to misophonia symptom-provocation or universally unpleasant sounds. Participants’ misophonia sound sensitivity, and emotional behaviors towards trigger sounds were assessed using the Misophonia Questionnaire. Stronger emotional behavioral reactions to misophonia trigger sounds were significantly associated with the larger Stroop effect when participants were exposed to the misophonia trigger sounds, but not when they were exposed to the universally unpleasant sounds. This effect held when controlling for the personality trait of Neuroticism and for baseline levels of anxiety. Both elevated misophonia sound sensitivity and emotional behaviors towards trigger sounds significantly correlated with higher self-reported anxiety when performing the Stroop task. However, only elevated emotional behaviors towards trigger sounds were linked with higher anxiety levels at baseline, suggesting that people who experience stronger emotions and behavioral reactions to misophonia trigger sounds may have higher anxiety at a trait level. Limitations and future directions are discussed.

## Introduction

Auditory sensitivity, sensory responsivity, and Selective Sound Sensitivity Syndrome are all terms that describe overlapping symptomology in a disorder that has recently been identified as misophonia, meaning “the hatred of sound” [1]. The general symptoms of misophonia include high sensitivity to everyday noises, such as coughing, sniffing, throat-clearing, and pen-tapping, which often result in a strong emotional response and, in some cases, an aggressive physical response [2]. Some people have also reported sensitivity to visual stimuli, where watching someone eat caused a similar emotional response [3]. In a large-scale study of over 300 people with misophonia, it was determined that their symptoms begin early in life, may be hereditary in nature, and increase over time [4].

Although the disorder has not been officially classified in the Diagnostic Statistical Manual of Mental Disorders [5], diagnostic criteria have been proposed [3]. Diagnostic criteria were determined by a series of scales assessing common symptoms and common response behaviors of participants who indicated misophonic symptoms. It was found that 81% of respondents were triggered by sounds made during eating, such as chewing, swallowing, and smacking of the lips, 63% were bothered by loud breathing sounds, and 59% were bothered by pen-clicking and keyboard-typing sounds. If participants generated the symptom-provocation triggers themselves or triggers were generated by animals, participants did not experience the same distress response. In the same study, 60% of participants reported feeling irritation in response to a trigger, while 41% reported feeling disgust. These feelings eventually translated to anger as the sound continued. Thirty percent of respondents indicated that they had been verbally aggressive at times in response to a trigger, while 16% reported physical aggression. The most severe cases were 12% that reported hitting a partner in response to them making the bothersome sounds.

Recent neuroimaging investigations confirm that there may be a neural basis for misophonia. People with misophonia symptomatology showed greater activity in the anterior insular cortex (AIC) to the misophonia-related cues compared to the neutral or universally unpleasant cues, compared to the control group [6]. The AIC is an integral region of the salience network, which is typically implicated in managing attention and devoting it to relevant stimuli for a specific task or function [7]. The AIC is also involved in emotional processing, specifically in the emotion of anger [8]. The same study reported increased functional connectivity between the AIC and the ventromedial prefrontal cortex, posteromedial cortex, hippocampus, and amygdala in the misophonia group, suggesting that there are neural underpinnings for the salience of and the emotional response to misophonia-related cues. Convergent evidence for the involvement of the salience network in misophonia comes from a more recent study, in which the right anterior cingulate cortex (ACC) and right insula were activated to a greater extent in people with misophonia (vs. without) in response to misophonic cues [9]. Together, these results indicate that people with misophonia may attribute more salience to the misophonic-related cues.

While many are skeptical of the disorder, those suffering from it are eager for answers and solutions to its effects. The intense anger and desire to react aggressively makes people with misophonia feel that they lack self-control, and they oftentimes report feeling guilty, which can result in anxiety and depression [3]. People with misophonia also report feelings of stress or discomfort when anticipating misophonic stimuli, and often turn to avoidance as a way to cope, which negatively reinforces the effects of the triggers. Some have even reported cutting off relationships with family or friends to avoid the extreme distress caused by the sounds [10].

Although people with misophonia self-report feelings of decreased control in response to the trigger sounds, little empirical evidence exists examining the effects of misophonia trigger sounds on cognitive control. Cognitive control encompasses a set of processes that are involved in generating and maintaining appropriate task goals and suppressing task goals that are no longer relevant [11]. Cognitive control is suggested to be a limited resource that can be depleted, where people lose resilience over time during a task that requires inhibition of competing processes [12, 13]. Tasks requiring cognitive control have been shown to activate dorsolateral prefrontal cortex (DLPFC) and the anterior cingulate cortex ACC [14].

Because people with misophonia symptoms report considerable distress in response to misophonia symptom-provocation triggers, it is likely that their attentional demands are greater, and thus processing, and possible attempted inhibition of undesired responses, may interfere with an unrelated task that requires cognitive control. From the limited resource perspective, misophonia triggers may deplete cognitive control resources to a greater degree in people with misophonia symptomatology.

Misophonia has generally been discussed as a clinical disorder, and much of the existing empirical evidence is based on investigations of clinical populations [6]. A study of the individual misophonia symptom frequencies, however, has revealed that misophonia symptomatology is presented on a continuum in a non-clinical sample, with 23.4% of participants reporting being “sometimes” sensitive to certain trigger cues, and 19.9% of participants endorsed having clinically significant misophonia symptoms [15].

Thus, the goal of the present study was to examine individual differences in the degree to which misophonia trigger cues have an effect on people’s cognitive control and consequent states of anxiety in a non-clinical sample. Cognitive control was assessed with a Stroop task, in which cognitive control is inferred in the process of setting up an attentional focus towards the ink color of a presented word, and away from the prepotent tendency to read the word, with the larger Stroop effect indicating worse cognitive control.

Critically, in a within-subject design, participants performed the Stroop task while being exposed to the misophonia symptom-provocation sounds, or to the control sounds (universally unpleasant sounds). A similar procedure was used in a study that investigated the effects of music on cognitive control [16]. Participants completed the Misophonia Questionnaire MQ [15] to examine individual differences in misophonia. The MQ is comprised of two factors: the *Misophonia sound sensitivity factor* surveys people’s general sensitivity to a variety of sounds that are thought to represent misophonia trigger cues (e.g., people eating, making nasal sounds, or repetitive tapping); and the *Emotional behaviors towards misophonia trigger sounds factor* surveys people’s specific responses to the sounds, including their emotional (e.g., becoming sad or depressed) and behavioral reactions (e.g., leaving the room or becoming physically aggressive).

Inhibition of unwanted responses, such as those surveyed by the behavioral reactions sub-factor of the MQ, arguably relies on a stronger engagement of cognitive control compared to only experiencing sensitivity to misophonia triggers without the urge to engage in corrective behaviors. Thus we expected that there would be a significant association between elevated emotional behaviors towards trigger sounds and the larger Stroop effect when exposed to the misophonia trigger sounds.

### Self-reported anxiety

Prior literature shows that people with misophonia symptomatology report anxiety in response to misophonia trigger sounds [17, 18, 19]. In the present study, we used the State-Trait Anxiety Inventory STAI [20] in which participants reported baseline anxiety, as well as state anxiety levels after each sound condition. Based on prior literature showing that misophonia in general is linked with negative affect [9], we hypothesized that there would be a significant link between higher misophonia symptomatology and elevated anxiety levels after exposure to both the misophonia trigger sounds, and to the universally unpleasant sounds.

### Neuroticism

Negative emotions are frequently expressed by people high in the personality trait of Neuroticism. Specifically, people high in neuroticism are more likely to report somatic complaints [21] and daily negative mood [22]. To control for potential contribution of neuroticism to the individual differences in self-reported misophonia, a measure of neuroticism was included as a control variable in the present study.

## Methods

### Participants

Seventy-nine (50 females, 28 males, and one “other”) participants took part in the present study (mean age = 21.94, *SD* = 5.80). Participants were university students, and received up to $13 for their participation. Sixty-three percent were Caucasian, 15% Latino/Latina, 8% Asian/Asian American, 4% African American, 4% American Indian/Native American/Alaska Native, 4% multi-racial, and 2% Middle Eastern/Arab. The study was approved by the University of Arkansas Institutional Review Board, and all participants signed an informed consent prior to participating.

### Measures

The *Stroop task* was used as a measure of cognitive control. Participants used three designated keys on the computer keyboard to indicate the color of a presented word on a computer screen. The task consisted of congruent trials, in which the color that the word was written in was the same color as the actual word (e.g. the word “red” written in red color); the incongruent trials, in which the color that the word was written in was different from the actual word (e.g. the word “red” written in green color); and neutral trials, in which a series of x’s were substituted for a word (e.g. “xxxx” written in green color). On all trials, participants were asked to name the color in which the word was written in, rather than reading the actual word. Participants completed 30 practice trials with no sounds before proceeding to two blocks of the test trials, which consisted of 108 trials per block (36 neutral, 36 congruent, and 36 incongruent). The inter-trial interval was jittered at 350, 500, and 750 ms. Block 1 was accompanied with continuously played misophonia trigger sounds, and Block 2 was accompanied with the universally unpleasant sounds. The order of the blocks was randomized across participants. Only trials (within-subjects) with reaction times (RTs) within the 2.5 *SD*s from the mean were analyzed. Only correct trials were included in the analyses. The dependent measure was the Stroop effect, calculated as the difference between response times to the incongruent and congruent trials, with the larger Stroop effect indicating worse cognitive control.

The task was presented on a Windows PC desktop. Four participants were excluded from analyses based on their Stroop task performance: Two participants had a high number of errors on the Stroop task (more than 2.5 *SD* from the mean), one participant’s reaction times were slower than 2.5 *SD* from the mean, and one participant’s Stroop effect was 2.5 *SD* above the mean.

*Audio recordings* [6] were used for the two sound conditions of the Stroop task. The audio recordings contained misophonia trigger sounds (e.g. eating sounds, breathing and nasal sounds, etc.), and universally unpleasant sounds (e.g. a baby crying, a person screaming; see S1 Appendix for a full list of the sounds). Each condition contained 14 different sounds, and each sound lasted 15 seconds. The sounds played at the same volume (70 dB), continuously presented one after another without any time intervals between the sounds. The sounds played in the same order for each participant.

*The Misophonia Questionnaire* MQ [15] was used to assess individual differences in misophonia sound sensitivity, and emotional behaviors towards the trigger sounds. The MQ consists of 17 items, and is comprised of two factors: The misophonia sound sensitivity factor surveys how sensitive people are to a variety of sounds in comparison to others. Some examples of the sounds are: People eating (e.g. chewing, swallowing, lip smacking, slurping, etc.), people making nasal sounds (e.g. inhaling, exhaling, sniffing, etc.), and repetitive tapping. The emotional behaviors towards trigger sounds factor surveys people’s reactions in response to the sounds, such as leaving the room, becoming sad or depressed, covering ears, and becoming verbally or physically aggressive. Responses are made on a Likert-type scale from “not at all true” (0) to “always true” (4), with responses summed within each sub-scale, as well as across the subscales to form the total MQ score. The MQ developers report good internal consistency, and convergent and discriminant validity [15]. In the current study internal consistency was *α* = .88 for the total scale, *α* = .80 for the misophonia sound sensitivity factor, and *α* = .83 for the misophonia emotional behaviors towards trigger sounds factor. Mean total MQ score was 22.97 (*SD* = 11.32, min = 2, max = 57), mean MQ sound sensitivity score was 11.47 (*SD* = 6.15, min = 0, max = 28), mean MQ emotional behaviors towards trigger sounds was 11.51 (*SD* = 6.48, min = 0, max = 29).

*The State-Trait Anxiety Inventory* STAI [20] consists of 20 statements that are used to assess participants’ current or “state” anxiety at the time they complete the survey. All answers are measured on a Likert-type scale, with the responses for the state anxiety ranging from “not at all” (1) to “very much so” (4). Examples of state anxiety items include “I am tense;” “I am worried,” and “I feel calm;” “I feel secure” (reverse-scored). Participants completed the survey at the beginning of the session (baseline), and once after each of the two Stroop task blocks.

*The Big 5 Neuroticism Scale* [23] assesses how well ten statements describes participants, using responses that range from “very inaccurate” (1) to “very accurate” (5). Examples of the statements are “I am easily disturbed,” and “I get upset easily.” Internal consistency for the Neuroticism scale in the current study was *α* = .89. Mean Neuroticism score was 2.56 (*SD* = .84, min = 1.0, max = 4.9)

### Procedure

Before starting the Stroop task, participants completed the STAI [20] to indicate their baseline anxiety levels. Participants then completed practice blocks of the Stroop task before starting on the experimental blocks. Before starting the task, participants were warned that some of the sounds may be unpleasant. After each block, participants were prompted to fill out the STAI survey indicating how they were feeling at that moment. After completion of the Stroop task, participants filled out a series of questionnaires, including the MQ [15], the Big 5 Neuroticism scale [23], and demographic information. Finally, participants watched a one-minute video of young children and dogs playing to alleviate any distress they may have experienced during the Stroop task. The session lasted approximately 60 minutes.

**Analytical strategy.** All analyses were conducted using [24] software. First, differences between the misophonia trigger and the universally unpleasant sound conditions in the overall RTs and in the Stroop effect were examined in the within-subject *t*-tests. Next, a Pearson correlation was performed to examine the associations between emotional behaviors toward misophonia trigger sounds and the Stroop effect when exposed to the misophonia trigger sounds. Additional correlations were performed for comprehensiveness, although we didn’t have specific predictions regarding these correlations. These analyses were followed by a linear regression model, with the Stroop effect when exposed to the misophonia trigger sounds as the dependent variable, to examine the association with the emotional behaviors toward misophonia trigger sounds while controlling for the personality trait of Neuroticism. Finally, a series of correlations and linear regressions were performed to examine the link between self-reported anxiety after the Stroop blocks that were paired with the misophonia trigger sounds and the universally unpleasant sounds, and the scores on the MQ sound sensitivity and MQ universally unpleasant sounds. Because four individual hypotheses were tested, the significance cutoff was set to .013 (= .05/4).

## Results

### Overall Stroop effect

A paired-sample *t*-test indicated that overall, participants responded slower on the incongruent (M = 747 ms, *SD* = 185) than on the congruent trials (M = 638 ms, *SD* = 134), demonstrating a reliable 109 ms Stroop effect, *t*(74) = 13.41, *p* < .001, Cohen’s *d* = 1.53.

Overall, participants made errors on 2.67% of trials (*SD* = 1.77), and made more errors on incongruent (M = 3.67%, *SD* = 2.65) than on the congruent trials (M = 2.70%, *SD* = 1.87), *t*(74) = 3.00, *p* = .004, Cohen’s *d* = .35.

### Reaction time differences between the misophonia and the universally unpleasant sound conditions

A paired-sample *t*-test indicated that overall participants responded marginally slower when exposed to the misophonia sounds (M = 688 ms, *SD* = 166) than when exposed to the universally unpleasant sounds (M = 676 ms, *SD* = 150), *t*(74) = 1.97, *p* = .053, Cohen’s *d* = .21. Error rates did not statistically differ in the misophonia (M = 2.71%, *SD* = 1.99) and the universally unpleasant sound conditions (M = 2.75%, *SD* = 2.14), *t*(74) = 0.16, *p* = .87, Cohen’s *d* = .02.

A paired-sample *t*-test indicated that the Stroop effect did not significantly differ in the misophonia (M = 107.40 ms, *SD* = 80.22) and the universally unpleasant sound conditions (M = 100.08 ms, *SD* = 73.84), *t(*74) = .99, *p* = .33, Cohen’s *d* = .12.

### Individual differences in misophonia and the Stroop task

**Fig 1:**
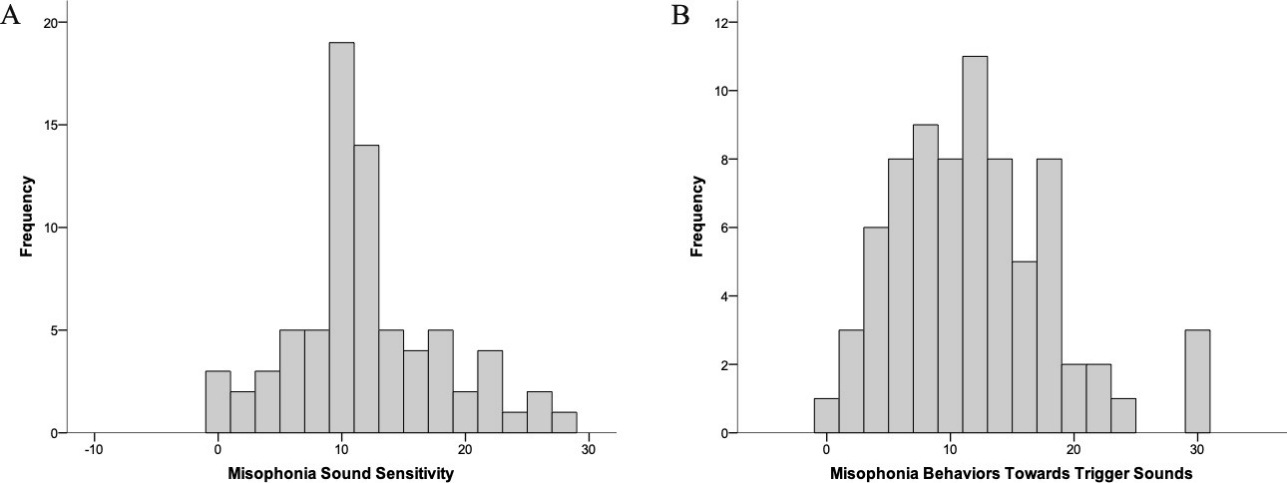
Distribution of Scores for MQ Sound Sensitivity (1a) and MQ Emotional Behaviors Towards Trigger Sounds (1b).

The distributions of scores for MQ sound sensitivity and MQ emotional behaviors towards trigger sounds are depicted in Fig 1. As can be seen from the figures, misophonia symptomatology presents on a continuum in a non-clinical sample, replicating results from [15].

**Fig 2:**
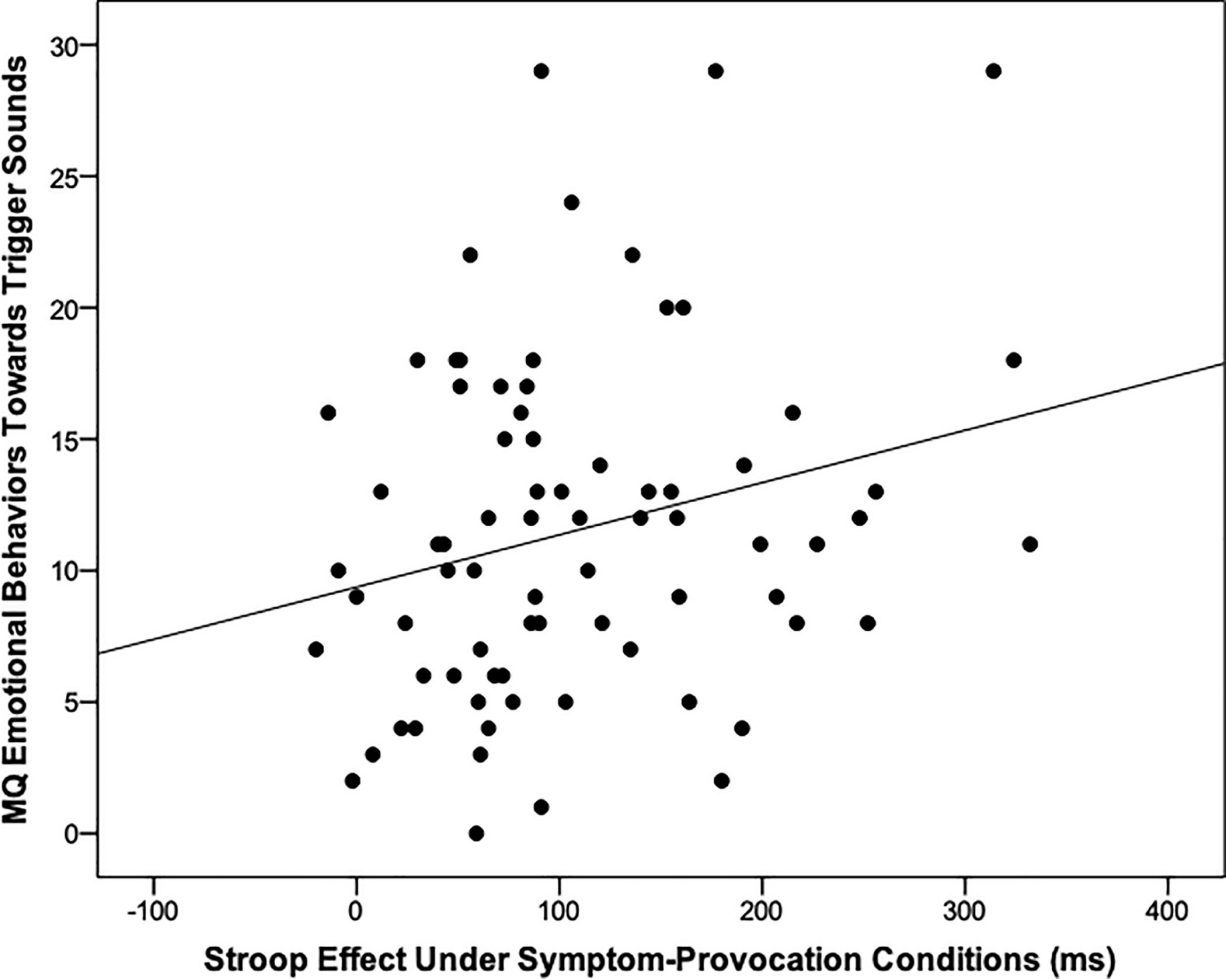
Pearson correlation between Misophonia Questionnaire (MQ) emotional behaviors to trigger sounds and the Stroop effect while exposed to the misophonia trigger sounds.

As predicted, emotional behaviors towards misophonia trigger sounds (*r* = .25, *p* = .03) were significantly associated with the larger Stroop effect when participants were exposed to the misophonia trigger sounds (Table 1; Fig 2), suggesting that people who experience stronger emotions and act out in misophonia symptom-provocation circumstances may experience stronger effects on their cognitive control. Critically, in a linear regression controlling for the personality trait of Neuroticism, emotional behaviors towards misophonia trigger sounds remained a significant predictor of a larger Stroop effect when exposed to the misophonia trigger sounds (Table 2).

**Table 1:** Zero-order correlations among MQ sound sensitivity, MQ emotional behaviors towards trigger sounds, MQ total score, and performance on the Stroop task.

	1	2	3	4	5	6	7
1. MQ Sound Sensitivity	--	.61	.90	.18	.09	.27	.18
2. MQ Emotional Behaviors to Trigger Sounds		--	.90	.25*	.17	.27	.23
3. MQ total			--	.24	.14	.30	.23
4. Stroop Effect RT—Misophonia Sounds				--	.24	.14	.30
5. Stroop Effect RT—Unpleasant Sounds					--	.45	.48
6. Stroop Total RT—Misophonia Sounds						--	.94
7. Stroop Total RT—Unpleasant Sounds							--
Mean	11.47	11.51	22.97	107.40	100.08	688.73	676.04
*SD*	6.15	6.48	11.32	80.22	73.84	165.75	149.80

MQ = Misophonia Questionnaire, RT = reaction times.

* *p* < .05

**Table 2:** Linear regression analysis predicting Stroop effect when exposed to misophonia sounds.

	B	SE	*ß*	*t*	*p*	95% CI
DV: Stroop Effect RT—Misophonia Sounds						
MQ Emotional Behavior	3.40	1.54	.27	2.21	.03	[.33; 6.47]
Neuroticism	-6.76	11.83	-.07	-.57	.57	[-30.34; 16.81]

MQ = Misophonia Questionnaire, STAI = State-Trait Anxiety Inventory; RT = reaction times.

There was no significant association between the emotional behaviors towards misophonia trigger sounds and the Stroop effect in the universally unpleasant sound condition, *r* = .17, *p* = .15. Similarly, there was no significant association between the MQ sound sensitivity and the Stroop effect in either the misophonia trigger (*r* = .18, *p* = .12) nor in the universally unpleasant sound condition (*r* = .09, *p* = .46; Table 1).

Further, zero-order correlations indicated that both elevated sensitivity to sounds and emotional behaviors towards misophonia trigger sounds as measured by the MQ were significantly associated with slower reaction times on the Stroop task in the misophonia, but not in the unpleasant sounds condition (Table 1), suggesting that individual differences in misophonia symptomatology overall, including sound sensitivity, may be linked with slower processing speed under misophonia symptom-provocation circumstances.

Neuroticism showed significant associations with MQ sound sensitivity (*r* = .32, *p* = .005), and the MQ emotional behaviors to trigger sounds (*r* = .40, *p* < .001); however, neuroticism was not significantly associated with the Stroop effect, nor with the overall Stroop task RTs in neither the misophonia, nor in the universally unpleasant sound conditions, *p*s > .33.

#### Self-reported anxiety

A paired-sample *t*-test indicated no block order effects of the sound conditions, Block 1 mean = 1.94 (*SD* = .47), Block 2 mean = 1.98 (*SD* = .51), *t*(74) = 1.05, *p* = .30, Cohen’s *d* = .12, indicating that the order in which the blocks were presented did not have an effect on the reported anxiety levels. Overall, participants indicated higher levels of anxiety when exposed to the unpleasant sounds (M = 2.06, *SD* = .49) compared to when exposed to the misophonia sounds (M = 1.96, *SD* = .53), *t*(75) = 2.50, *p* = .02, Cohen’s *d* = .30, revealing that in general people felt more anxious listening to the universally unpleasant versus misophonia trigger sounds.

With regard to our hypotheses, individual differences in MQ misophonia sound sensitivity and MQ emotional behaviors towards trigger sounds were significantly associated with self-reported anxiety while being exposed to both misophonia and the universally unpleasant sounds (Table 3). These effects held in a linear regression after controlling for baseline anxiety levels (*p*s < .001), or for personality trait of Neuroticism, *p*s < .03).

**Table 3:** Zero-order correlations among MQ sound sensitivity, MQ emotional behaviors towards trigger sounds, MQ total score, and anxiety scores.

	1	2	3	4	5	6
1. MQ Sound Sensitivity	--	.61	.89	-.01	.36*	.29*
2. MQ Emotional Behavior to Trigger Sounds		--	.90	.25	.48*	.44*
3. MQ Total			--	.14	.46	.41
4. STAI Baseline				--	.46	.56
5. STAI Misophonia Sounds					--	.79
6. STAI Unpleasant Sounds						--

*p < .013

Higher emotional behaviors towards trigger sounds were significantly associated with elevated baseline anxiety (*r* = .25, *p* = .03), while there were no significant links between baseline anxiety and MQ total or MQ sound sensitivity (*p*s > .23).

## Discussion

The present study aimed to investigate the extent to which the severity of misophonia symptoms is linked with cognitive control under misophonia symptom-provocation circumstances in the general population sample. In a within-subject design, participants completed a Stroop task while being exposed to the misophonia trigger sounds and the universally unpleasant sounds. The Stroop effect–i.e., the interference effect associated with how much slower participants responded to the incongruent than to the congruent trials, was a measure of cognitive control. Results revealed that stronger misophonia symptomatology was associated with a larger Stroop effect when participants were exposed to the misophonia trigger sounds. This effect was unique to the emotional behaviors towards the trigger sounds, while misophonia sound sensitivity alone was not significantly associated with the Stroop effect. These results suggest that people who experience certain sensitivity to the misophonic sounds, but do not experience strong emotions or do not feel the need to engage in various corrective behaviors, may not experience decreased cognitive control in misophonia symptom-provocation circumstances. People who report engaging in specific responses to the sounds, including becoming sad or depressed, or leaving the room or becoming physically aggressive, appear to experience a reduction in their cognitive control in symptom-provocation circumstances.

Importantly, these results held after controlling for the personality trait of Neuroticism. While neuroticism correlated with both misophonia sound sensitivity and emotional behaviors to trigger sounds, our results show that reduced cognitive control in symptom-provocation circumstances is not a function of higher neuroticism, but is specific to elevated misophonia symptomatology.

Our results are novel in the sense that they provide the first account of functional depletion of cognitive control in people who are likely to engage in various behavioral coping strategies in misophonic situations. The results are in line with prior literature, which shows that people with misophonia often report feeling a lack of self-control and a desire to act out in misophonic situations [3]. Indeed, ACC, previously found to be implicated during viewing of misophonic video clips [9] is the same region that is involved in resolving conflict on paradigms like the Stroop task [12]. Considering that cognitive control is a limited resource, inhibition of undesirable behaviors in such circumstances may deplete cognitive control to a greater degree in people with elevated misophonia symptomatology. To our knowledge, this is the first study to demonstrate this effect in a non-clinical sample.

Further, participants with both elevated misophonia sound sensitivity and emotional behaviors to trigger sounds were overall slower on the Stroop task in the misophonia trigger condition, but not in the universally unpleasant sound condition, indicating that misophonia may be linked with overall decreased processing speed in symptom-provocation circumstances. This finding warrants further investigations. Although not predicted, the total MQ score was significantly associated with slower overall responses in the unpleasant sound condition, but this was not the case when examining the MQ two sub-factors separately. Future studies need to investigate whether misophonia symptomatology may be linked with slower processing speed in circumstances outside of the ones that are known to provoke misophonic symptoms.

Our results complement previous work that reported negative effects of misophonia sounds on learning in people with high misophonia sensitivity [25]. In this study, one misophonia trigger sound was used (i.e., chewing gum), compared to the absence of gum chewing (i.e., silence). Here, we employed a more suitable control condition (i.e., universally unpleasant sounds), and used 14 unique sounds per condition.

### Anxiety and misophonia

Both higher MQ sound sensitivity and higher MQ emotional behaviors to trigger sounds were significantly associated with elevated self-reported anxiety in both the misophonia and the universally unpleasant sound conditions, even after controlling for neuroticism. These results support literature showing that people with misophonia report severe anxiety in symptom-provocation circumstances [19], and exhibit elevated galvanic skin response–a physiological marker of arousal, in response to trigger sounds [6] and visual images [9]. In the current study we found that the severity of misophonia symptoms was significantly related to increased self-reported anxiety while exposed to both misophonia and universally unpleasant sounds, although the severity of misophonia symptoms was not linked with baseline anxiety reports. These results should be further examined in future studies.

### Limitations and future directions

Given the largely auditory triggers for misophonia, future investigations need to go beyond standard visual Stroop task and use an auditory Stroop task, which would allow for addressing questions about the seeming modality-specificity of the inducing stimuli. Further, although we report a link between misophonia symptomatology and self-reported anxiety, other emotions should be examined in future studies, including anger, disgust, and sadness. It has been noted that understanding the role of attention and cognitive control in misophonia could potentially lead closer to developing treatments and therapies for people affected by misophonia [17]. While the present study examined the link between the severity of misophonia symptoms and cognitive control, further studies are needed that evaluate the link between misophonia and other aspects of executive function, such as attention and working memory.

### Conclusion

Results from the present study provide evidence that misophonia may significantly deplete cognitive control when people with elevated misophonia symptomatology are placed in misophonia symptom-provocation circumstances. Even after controlling for the personality trait of Neuroticism, cognitive control was significantly reduced in people who reported elevated misophonia emotional behaviors to trigger sounds when they were exposed to such sounds. Further, both elevated misophonia sound sensitivity and emotional behaviors to trigger sounds were linked with overall slower speed of responses on the measure of cognitive control, specifically when exposed to the triggers. Finally, the severity of misophonia symptoms was significantly associated with higher self-reported anxiety in both symptom-provocation circumstances, and when exposed to universally unpleasant sounds. These results provide strong support that misophonia is associated with functional depletion of cognitive control under symptom-provocation circumstances, demonstrating the need for future studies.

## Supporting information

S1 Appendix (DOCX)
