# Supplementary material for: Severity of misophonia symptoms is associated with worse cognitive control when exposed to misophonia trigger sounds
Source: PLoS One. 2020 Jan 16;15(1):e0227118. doi: 10.1371/journal.pone.0227118 (PMC6964854; doi:10.1371/journal.pone.0227118)
Supplement: S1 Appendix — (DOCX) [file pone.0227118.s001.docx]

**Appendix**

*Misophonia Trigger Sounds*

apple crunching

breathing

coughing/sniffing

eating potato chips

cutlery sounds

eating salad with cutlery sounds

gulping water

packet rustling

slurping

slurping with eating

sniffing

soft chewing

eating sounds 1

eating sounds 2

*Unpleasant Sounds*

vomiting

one baby crying

multiple babies crying

belching/burping

bees buzzing

generic insects buzzing

dentist drill

alarm clock

female crying

female screaming

jack hammer

male crying

dogs barking

toddler crying
